# Correlational study on the sense of humor and positive mental health in mental health professionals

**DOI:** 10.3389/fpubh.2024.1445901

**Published:** 2024-11-05

**Authors:** Sergi Piñar-Rodríguez, Dolors Rodríguez-Martín, David Corcoles-Martínez, Diana Tolosa-Merlos, Miriam Leñero-Cirujano, Montse Puig-Llobet

**Affiliations:** ^1^PhD Program, Faculty of Nursing, University of Barcelona, Barcelona, Spain; ^2^Department of Fundamental and Clinical Nursing, Faculty of Nursing, University of Barcelona, Health Sciences Campus of Bellvitge, Barcelona, Spain; ^3^Mental Health Institute, Hospital del Mar, Barcelona, Spain; ^4^Department of Nursing, Faculty of Medicine, Autónoma University of Madrid, Gregorio Marañón Health Research Instituto (IiSGM), Madrid, Spain; ^5^Department of Public Health, Mental Health and Maternal-Infant Nursing, Faculty of Nursing, University of Barcelona, Barcelona, Spain

**Keywords:** correlational study, mental health care professionals, wit and humor as topic, positive mental health, therapeutic relationship

## Abstract

**Background:**

Mental health professionals require exceptional communication skills and the ability to maintain an empathetic and authentic attitude within the therapeutic relationship. It is crucial that they achieve an optimal balance of physical, mental, and social wellbeing to enhance their performance in this context. This necessity has sparked a growing interest in promoting mental health among these professionals by focusing on the evaluation of both Positive Mental Health and the Sense of Humor.

**Objective:**

To assess the level of sense of humor and positive mental health, and to analyse the relationships between the sense of humor construct, the positive mental health construct, and the sociodemographic, occupational, and educational characteristics of mental health professionals who care for patients in hospital and community settings.

**Methods:**

An observational, descriptive, and cross-sectional with a non-experimental quantitative approach study has been carried out. The study was conducted involving 130 mental health care professionals. Levels of sense of humor and positive mental health were evaluated using authenticated questionnaires, while the relationship between these two constructs and the sociodemographic, occupational, and training characteristics of health workers were analyzed following STROBE guidelines.

**Results:**

The study involved 130 professionals, predominantly women (71.5%), with an average age of 41.4 years. The majority were nurses (45.4%) with varied work experience and educational levels. Regarding the PMHQ questionnaire, an average score of 102.6 was achieved in the general evaluation, showing an insignificant relationship with sociodemographic and occupational variables. However, a significant trend regarding age and autonomy was noted. On the other hand, the Multidimensional Sense of Humor Questionnaire produced an average score of 67.3, also without significant correlations with the variables under scrutiny. Although no positive relationships were found between the general scores of Positive Mental Health Questionnaire and Multidimensional Sense of Humor Questionnaire, a positive correlation emerged between the use of humor and situational control. In summary, the findings suggest that the level of autonomy and the use of humor may be associated with specific sociodemographic and occupational characteristics, although the precise relationship remains complex and requires further research.

## Introduction

Mental health professionals require excellent communication skills, as well as the ability to maintain an empathetic, humane, and sincere attitude within the therapeutic relationship (1). These factors enable professionals to approach their daily work in the best possible way, aiming to strengthen therapeutic bonds (2). Patients with mental health diagnoses rely on various resources from medical, social, and family care settings (3), and numerous studies confirm that many healthcare professionals exhibit signs of high stress levels due to the complexity of the care they must provide, as well as other aspects related to the profession itself and its management, all of which can cause frustration and low self-esteem at work (4–6).

There is a growing interest in promoting mental health among professionals by assessing levels of both Positive Mental Health (PMH) (7–9) and Sense of Humor (SH) (10). Professionals must achieve the right balance of physical, mental, and social wellbeing to perform their best within the therapeutic relationship (11). They need to be able to identify any factors that may prevent patients from adapting to their environment. Families of patients diagnosed with mental health disorders often feel emotionally overwhelmed by the complexity of the illness, and therefore, professionals must be able to intervene empathetically in a close and authentic manner (12, 13).

In this context, in 1999, Lluch proposed a multifactorial model of PMH, adapting Jahoda's mental health model, which established six factors as constituents of the PMH construct: personal satisfaction (F1), prosocial attitude (F2), self-control (F3), autonomy (F4), problem-solving and self-actualization (F5), and interpersonal relationship skills (F6). This model also confirms the dynamic characteristic of the PMH construct, where both positive and negative thoughts fluctuate, as well as its foundation in a holistic view of health by understanding the close link between physical and mental health. Promoting PMH is a crucial part of every nursing intervention and is conducted in both health and disease processes (14). Similarly, the SH construct holds significant prominence within the field of study of emotions and positive psychology. Regarding the latter, the SH construct highlights the psychological benefits of feelings such as joy, wellbeing, satisfaction, a sense of activation, and calm. SH is considered a strength that provokes pleasant, positive, and rewarding feelings (15).

One of the strengths of SH is its ability to facilitate communication with patients, specifically mental health patients. In addition to being present in a wide variety of social situations, SH is both a resource and a necessity for many people, and for those working in nursing, SH provides humor as well as meaning. For example, mental health professionals can benefit from SH as it allows them to be more competent and maintain more satisfying and authentic relationships both with patients and families (10, 16). The favorable effects of humor on health have been studied in various healthcare settings such as pediatrics, oncology, palliative care, and even for patients with dementia and their caregivers (17–21). Rosemary Parse applied her Human Development Theory in a study on humor and laughter in patient interaction and observed its impact on health (21, 22).

Although there are several studies investigating the fields of SH and PMH, it is noteworthy that to date, both constructs have not been explored together. Mental health professionals are a crucial element in the therapeutic link between patients and their families and are responsible for maintaining a more humane relationship focused on holistic care. The main hypothesis of the study is that there could be differences in positive mental health and sense of humor among professionals who care for mentally ill patients. Building on this hypothesis, the aim of the study focused on evaluating levels of sense of humor and positive mental health while analyzing the relationships between the SH and PMH constructs and the sociodemographic, occupational, and training characteristics of mental health care professionals providing care in both hospital and community settings.

## Methodology

### Study design

An observational, descriptive, and cross-sectional with a non-experimental quantitative approach study has been carried out.

### Participants

The study involved 130 healthcare professionals recruited from three mental health service centers. There are three psychiatric hospital centers, two of them in the city of Barcelona, and one in the city of Santa Coloma. All three centers belong to Parc de Salut Mar. The professionals could come from both, the hospitalization area or from outpatient consultation. Participants joined progressively from September 2022 to January 2023, all volunteering and meeting the required inclusion criteria. All participants were employed professionals with a minimum of 1 year of experience in mental health. Those on leave, including sick leave, as well as professionals on external rotation from other health institutions, were excluded. Participants received a document containing relevant and necessary information for participation in the study, and researchers collecting data informed participants about the nature of the study, as well as any risks and benefits, thus resolving any doubts or questions that might arise during the completion of the questionnaires.

### Procedures

All participants were asked to fulfill two scales: The Positive Mental Health Questionnaire (PMHQ) and the Multidimensional Sense of Humor Questionnaire (MSHQ).

### Variables

The following variables were collected for each participant: (a) age and sex; (b) years of experience in mental health (< 10, 11–20, more than 20), professional category (psychiatrist, nurse, Nursing Care Technicians –TCAI-, other), type of contract (permanent, temporary, substitute, residential); and (c) training (postgraduate training, communication skills training).

### Instruments

The PMHQ (23) and the MSHQ (24) were administered to all participants. Both scales have been previously validated. The PMHQ includes 39 items unevenly distributed across six factors: F1, Personal Satisfaction; F2, Prosocial Attitudes; F3, Self-Control; F4, Autonomy; F5, Problem Solving and Self-improvement; F6, Interpersonal Relationship Skills (Table 1). Items are phrased as positive or negative statements and each response is scored on a scale of 1–4 based on frequency: always/almost always (4), frequently (3), sometimes (2), and never/almost never (1). Scores allowed for a single PMH value and specific values for each factor. The questionnaire was originally validated within a population of nursing students with a sample size of *n* = 387. Psychometric values were favorable: reliability levels with Cronbach's alpha rated between 0.89 and 0.90, and a test-retest correlation of 0.85. Principal component analysis highlighted six factors explaining 46% of total variance, and the factor loading for all items was >0.40 (Table 1). The MSHQ consists of 24 items, 18 positive (1–3, 5–7, 9, 10, 12, 14–16, 18, 19, 21–24) and 6 negative (4, 8, 11, 13, 17, 20) to minimize response bias. The items are distributed unevenly across three factors: F1, Personal competence in humor; F2, Humor as a mechanism to control a situation; and F3, Social evaluation and general attitude toward humor. Positive items are scored on a five-point Likert scale from Strongly Disagree (0) to Strongly Agree (4), and negative items in reverse. The lowest possible score is zero and the highest 96, with an average score of 64.45 and a standard deviation of 11.83. Alpha results range from 0.87 to 0.88 (Table 1).

**Table 1:** Item distribution by factors, maximum and minimum values of each factor, and overall scores of each questionnaire.

**Factors**	**Items**	**Minimum/Maximum**
**Positive Mental Health Questionnaire (PMHQ)**		
F1: Personal satisfaction	4^*^,6, 7, 12, 14, 31, 38, 39	8–32
F2: Prosocial attitude	1, 3, 23^*^, 25^*^, 37^*^	5–20
F3: Self-control	2, 5^*^, 21^*^, 22^*^, 26^*^	5–20
F4: Autonomy	10, 13, 19, 33, 34	5–20
F5: Problem solving and self-realization	15^*^, 16^*^, 17^*^, 27^*^, 28^*^, 29^*^, 32^*^, 35^*^, 36^*^	9–36
F6: Interpersonal relationship skills	8, 9, 11^*^, 18^*^, 20^*^, 24, 30	7–28
PMH total	1–39	39–156
**Multidimensional Sense of Humor Questionnaire (MSHQ)**		
F1: Personal competence or ability to use humor	1^*^, 3^*^, 5^*^, 6^*^, 7^*^, 9^*^, 12^*^, 15^*^, 18^*^, 21^*^, 23^*^, 24^*^	0–48
F2: Humor as a mechanism for controlling the situation	2^*^, 10^*^, 14^*^, 16^*^, 19^*^, 20, 22^*^	0–28
F3: Social appraisal and attitudes toward humor	4, 8, 11, 13, 17	0–20
MSHS total	1–24	0–96

^*^Positive items.

### Statistical analysis

A descriptive analysis of all variables in the study was performed and analyzed using absolute and relative frequencies for qualitative variables and measures of central tendency (average mean) and measures of dispersion (standard deviation or SD) for quantitative variables. The average means of a normally distributed numerical variable were compared using the Student's *t*-test when involving two groups and ANOVA for more than two groups, while Pearson's correlation was used to compare two numerical variables. The work was conducted with a 95% confidence level, and the difference between variables was considered significant when the *p*-value (degree of significance) was less than or equal to 0.05. Data were analyzed using the SPSS statistical package Version 24 (25). STROBE guidelines were followed for data analysis (26).

## Results

### Description of sociodemographic, occupational, and training results

A total of 130 professionals participated in the study: 93 women (71.5%) and 37 men (28.5%), with an average age of 41.4 years (SD: 11.7). Of the sample, 45.4% were nurses, 27.7% were TCAI, 16.9% were psychiatrists, and 10% were from other areas.

41.5% of the sample had < 10 years of work experience, 28.5% had 11–20 years of experience, and 30% had more than 20 years of experience. Of the total, a large percentage had a permanent contract (63.8%), while 15.4% had a temporary contract, 11.5% were on a residential contract, and 9.2% on a substitute contract. Although 62.3% of the sample did not have specific postgraduate training in mental health, 37.7% did, and a large percentage of the sample (82.3%) did not have postgraduate training in communication skills, with only 17.7% having such training (Table 2).

**Table 2:** Study population characteristics.

**(*n* = 130)**	***n***	**(%)**
Age (in years)	41.4 (^*^SD 11.7)	
**Sex**		
Female	93	71.5
Male	37	28.5
**Specialty**		
Psychiatrist	22	16.9
Nurse	59	45.4
Nursing assistant	36	27.7
Others	13	10
Work experience in mental health	54	41.5
< 10 years	37	28.5
11–20 years	39	30
**More than 20 years**		
Type of employment contract	20	15.4
Permanent	12	9.2
Temporary contract	15	11.5
**Substitute**		
Resident	49	37.7
**Postgraduate training in mental health**		
Yes	23	17.7
No	107	82.3

SD, standard deviation; *n*, sample; %, percentage.

### Positive mental health

In evaluating the PMHQ, an average total score of 102.6 was obtained (SD: 7.4). Total test scores ranged from 89 to a maximum of 135 (Table 3). Regarding the relationship between the PMHQ and sociodemographic, occupational, and training variables (Table 4). None of the correlations were statistically significant. However, a strong trend suggesting significance was observed.

**Table 3:** Descriptors of the PMHQ and MSHQ.

**Questionnarie**	**Minimum**	**Maximum**	**SD**	**Mean**
**MSHS**				
F1: Personal competence or ability to use humor	10	44	6.5	29.7
F2: Humor as a mechanism for controlling the situation	14	28	3	22.1
F3: Social appraisal and attitudes toward humor	9	20	2.5	15.4
MSHS Total	42	91	9.9	67.3
**PMHQ**				
F1: Personal satisfaction	22	31	1.7	27.6
F2: Prosocial attitude	9	16	1.3	11.8
F3: Self-control	7	20	2.2	10.6
F4: Autonomy	11	20	1.9	17,3
F5: Problem solving and self-realization	9	34	3.5	14.4
F6: Interpersonal relationship skills	15	49	3.6	20.7
PMHQ total	89	135	7.4	102.6

SD, standard deviation; PMHQ, Positive Mental Health Questionnaire; MSHQ, Multidimensional Sense of Humor Questionnaire.

**Table 4:** Sociodemographic, occupational, and training variables in the PMHQ.

			**Factor 1**		**Factor 2**		**Factor 3**		**Factor 4**		**Factor 5**		**Factor 6**		**PMH total**	
			**Personal satisfaction**		**Prosocial attitude**		**Self-control**		**Autonomy**		**Problem solving and self-realization**		**Interpersonal relationship skills**		
	***n***	**%**	**Mean (SD)**	***p***	**Mean (SD)**	***p***	**Mean (SD)**	***p***	**Mean (SD)**	***p***	**Mean (SD)**	***p***	**Mean (SD)**	***p***	**Mean (SD)**	***p***
Age (in years)	41.4 (DE 11.7)		*r* = −0.025	0.774	*r* = −0.016	0.858	*r* = −0.077	0.384	*r* = 0.190	0.030^*^	*r* = −0.042	0.635	*r* = 0.045	0.613	*r* = 0.024	0.786
**Gender**																
Female	93	71.5	27.6 (1.7)	0.356	11.7 (1.2)	0.276	10.7 (2.4)	0.584	17.1 (1.9)	0.204	14.3 (3.7)	0.454	20.9 (4.8)	0.655	102.6 (7.9)	0.516
Male	37	28.5	27.8 (2.0)		12.0 (1.4)		10.3 (1.7)		17.6 (2.0)		14.5 (2.9)		21.0 (3.9)		103.4 (7.4)	
**Specialty**																
Psychiatrist	22	16.9	27.7 (1.4)	0.901	11.6 (1.3)	0.844	11.2 (2.0)	0.408	16.6 (2.3)	0.021^*^	15.2 (3.7)	0.789	20.7 (1.6)	0.292	103.3 (7.2)	0.972
Nurse	59	45.4	27.5 (2.0)		11.8 (1.5)		10.7 (2.4)		17.1 (1.5)		14.3 (4.0)		20.6 (4.6)		102.3 (7.8)	
Nursing assistant	36	27.7	28.0 (1.3)		11.8 (1.1)		10.2 (1.9)		18.0 (2.0)		14.1 (2.6)		21.8 (6.0)		104.1 (8.5)	
Other professionals	13	10	27.4 (2.0)		11.9 (0.9)		10.5 (2.4)		16.8 (2.1)		14.1 (2.8)		20.3 (2.6)		101.3 (6.8)	
**Work experience in mental health**																
< 10 years	54	41.5	27.6 (1.7)	0.925	11.8 (1.2)	0.880	11.1 (2.2)	0.010^*^	16.8 (2.0)	0.048^*^	14.8 (3.3)	0.097^*^	20.8 (5.6)	0.160	103.2 (8.2)	0.006^*^
11–20 years	37	28.5	27.8 (1.7)		11.8 (1.3)		9.7 (1.7)		17.2 (1.5)		13.2 (2.4)		20.5 (1.4)		100.7 (3.9)	
More than 20 years	39	30	27.6 (1.9)		11.8 (1.4)		10.9 (2.4)		17.6 (2.0)		14.8 (4.3)		21.5 (5.0)		104.4 (9.4)	
**Type of contract**																
Permanent	83	63.8	27.7 (1.9)	0.663	11.8 (1.4)	0.504	10.2 (2.1)	0.011^*^	17.5 (1.8)	0.025^*^	14.0 (3.5)	0.024^*^	21.0 (3.69	0.063^*^	102.4 (7.4)	0.125
Temporary contract	20	15.4	27.9 (1.4)		11.7 (1.1)		11.7 (2.2)		16.8 (2.0)		14.9 (3.2)		19.6 (1.6)		102.6 (5.2)	
Substitute	12	9.2	27.2 (1.7)		11.6 (0.8)		11.0 (2.3)		17.4 (2.2)		13.0 (2.2)		21.5 (6.8)		101.9 (10.1)	
Resident	15	11.5	27.7 (1.4)		12.2 (1.4)		11.2 (2.2)		16.2 (1.4)		16.7 (3.6)		22.1 (8.6)		106.3 (10.3)	
**Postgraduate training**																
Yes	49	37.7	27.7 (2.0)	0.652	11.7 (1.4)	0.225	10.5 (2.3)	0.655	17.2 (1.8)	0.711	13.5 (2.7)	0.023^*^	20.1 (1.7)	0.292	100.9 (5.4)	0.067^*^
No	81	62.3	27.6 (1.6)		11.9 (1.2)		10.7 (2.2)		17.3 (2.0)		14.9 (3.8)		21.4 (5.6)		104.1 (8.7)	
**Communication skills training**																
Yes	23	17.7	27.5 (2.3)	0.910	12.1 (1.5)	0.375	11.5 (3.3)	0.370	16.9 (1.9)	0.346	15.5 (5.2)	0.276	20.8 (2.2)	0.606	104.6 (9.3)	0.218
No	107	82.3	27.7 (1.6)		11.7 (1.2)		10.4 (1.9)		17.3 (1.9)		14.1 (2.9)		21.0 (5.0)		102,5 (7.4)	

^*^p > 0.05; *r*, Pearson correlation; SD, standard deviation; *n*, sample; %, Percentage; PMHQ, Positive Mental Health Questionnaire.

Regarding the age of the subjects and F4 (Autonomy), the following was observed: *p* = 0.030, with an increase in individual criteria regardless of any increase in age. Regarding the relationship between professional category and F4 (Autonomy), psychiatrists and TCAI (an average score of 16.6 with SD: 2.3 and 18.0 with SD: 2.0, respectively) scored higher than nurses (an average score of 17.1 with SD: 1.5), achieving *p* = 0.021. It is noteworthy that years of experience in mental health care demonstrated a significantly high trend in F3: (*p* = 0.010) Self-Control, relating to the ability to maintain emotional balance and stress tolerance in the workplace, and in F5: (*p* = 0.097) Problem-Solving and Self-Improvement, relating to better analytical ability, adaptability to change, and decision-making. No statistically significant differences were found regarding other variables.

### Sense of humor

An average total score of 67.3 (SD: 9.9) was obtained, with total test scores ranging from 42 to a maximum of 91 (Table 3). The relationship between the MSHQ (both globally and for each factor) and sociodemographic, occupational, and training variables was analyzed using Pearson's *r* (Table 5). None of the correlations were statistically significant. However, a trend suggesting significance was observed.

**Table 5:** Sociodemographic, occupational, and training variables in the MSHQ.

			**Factor 1**		**Factor 2**		**Factor 3**		**MSHS total**	
			**Competence and ability to use humor**		**Humor as a means of controlling the situation**		**Social appraisal and attitudes toward humor**			
	***n***	**%**	**Mean (SD)**	***p***	**Mean (SD)**	***p***	**Mean (SD)**	***p***	**Mean (SD)**	***p***
Age (in years)	41.4 (DE 11.7)		*r* = 0.050	0.569	*r* = −0.083	0.3451	*r* = 0.094	0.285	*r* = 0.047	0.596
**Sex**										
Female	93	71.5	26.8 (6.0)	0.617	18.9 (2.0)	0.1221	15.5 (2.5)	0.767	53.4 (7.6)	0.983
Male	37	28.5	26.8 (6.5)		19.3 (1.6)		15.3 (2.8)		53.4 (6.9)	
**Professional specialty**										
Psychiatrist	22	16.9	28.6 (6.5)	0.193	19.3 (2.4)	0.9211	15.6 (2.3)	0.476	55.8 (6.2)	0.316
Nurse	59	45.4	26.5 (6.2)		19.0 (1.5)		15.5 (2.6)		52.9 (7.6)	
Nursing assistant	36	27.7	26.0 (5.9)		19.0 (2.1)		14.9 (2.9)		53.1 (7.2)	
Other professionals	13	10	27.1 (6.1)		19.0 (2.0)		16.1 (1.6)		52.6 (8.4)	
**Work experience in mental health**										
< 10 years	54	41.5	27.1 (5.4)	0.300	19.3 (1.9)	0.1481	15.1 (2.5)	0.490	53.8 (6.8)	0.242
11–20 years	37	28.5	25.1 (7.4)		18.5 (1.6)		15.7 (2.6)		51.5 (8.2)	
More than 20 years	39	30	28.0 (5.7)		19.2 (2.1)		15.6 (2.5)		54.7 (7.1)	
**Type of contract**										
Permanent	83	63.8	26.9 (6.2)	0.109	18.8 (1.8)	0.253	15.7 (2.6)	0.236	53.6 (7.4)	
Temporary contract	20	15.4	26.7 (6.0)		19.7 (2.1)		15.1 (2.5)		53.6 (7.3)	
Substitute	12	9.2	23.2 (6.2)		19.6 (2.2)		14.1 (3.2)		48.3 (8.1)	
Resident	15	11.5	29.2 (4.9)		19.0 (1.7)		15.4 (1.5)		55.8 (5.8)	
**Postgraduate training**										
Yes	49	37.7	25.9 (6.7)	0.228	18.7 (1.3)	0.1941	15.3 (2.6)	0.515	52.6 (8.1)	0.301
No	81	62.3	27.3 (5.7)		19.2 (2.2)		15.5 (2.5)		53.9 (6.9)	
**Training in communication skills**										
Yes	23	17.7	26.5 (6.7)	0.836	19.4 (2.6)	0.6671	15.8 (2.2)	0.449	53.4 (7.8)	0.566
No	107	82.3	26.8 (6.1)		19.0 (1.7)		15.4 (2.6)		53.4 (7.3)	

*r*, Pearson correlation; SD, standard deviation; *n*, sample; %, Percentage; MSHQ, Multidimensional Sense of Humor Questionnaire.

Regarding gender variability, it was observed that within factor F2 (the ability to use humor to control a situation), men scored higher on average than women (*p* = 0.122), while professional category variables within F1 (competence and ability to use humor) showed that psychiatrists scored higher on average than any other category (*p* = 0.193). Regarding the variable of work experience in mental health care, the average score within F2 of professionals with more experience was higher compared to those with less (*p* = 0.148), and concerning the variable of contract type within F1, the average score of professionals with permanent, temporary, or residential contracts was higher than those with substitute contracts (*p* = 0.109). No statistically significant differences were found regarding other variables.

### Relationship between positive mental health and sense of humor

**Figure 1:**
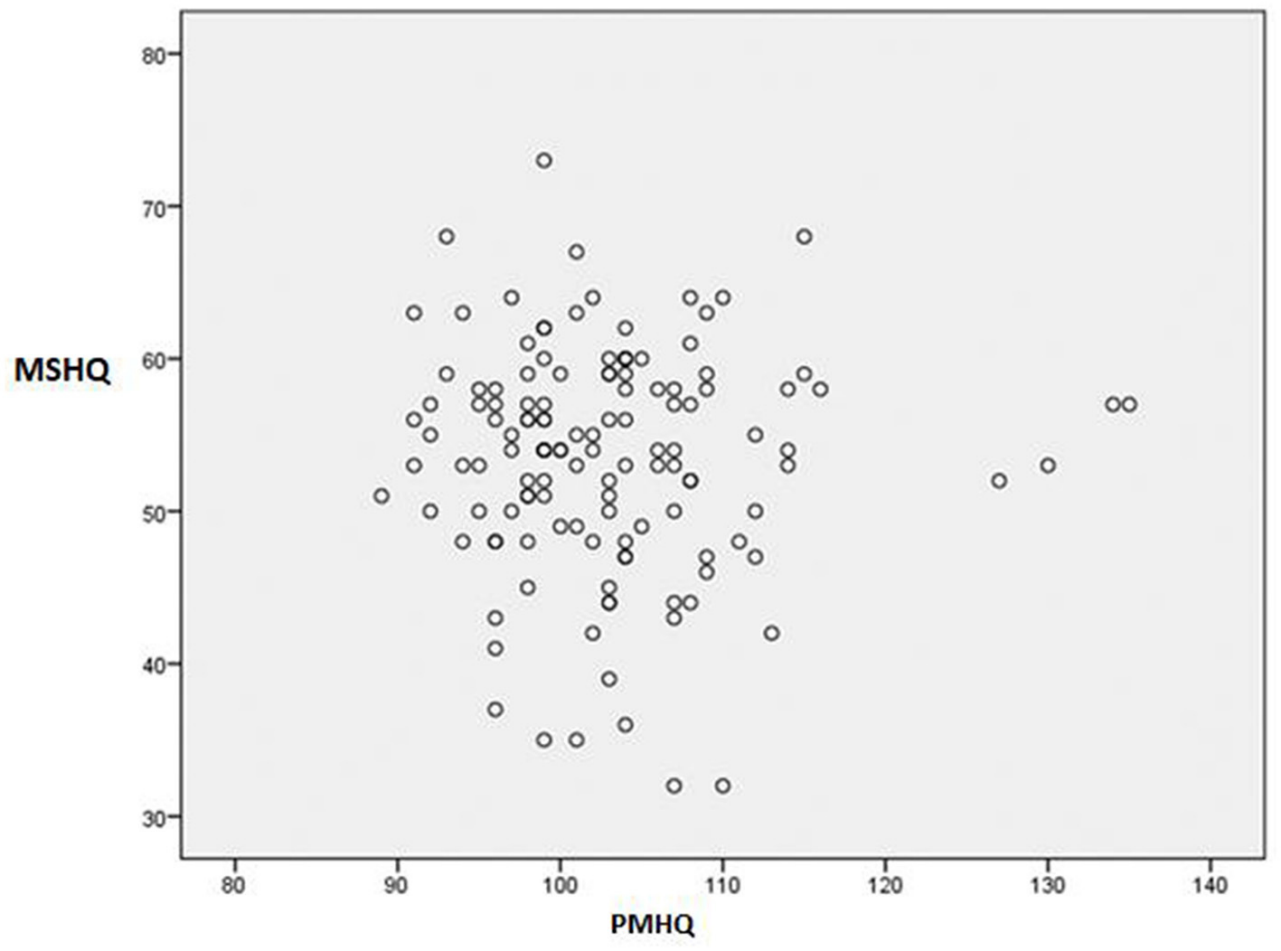
Analysis of the correlation of total scores of the PMHQ and MSHQ questionnaires. MSHQ, Multidimensional Sense of Humor Questionnarie; PMHQ, Positive Mental Health Questionnarie.

Regarding the general relationship between global scores in the PMHQ and MSHQ, the analysis revealed no positive relationship between the two (*r* = −0.017; *p* = 0.844). However, despite no positive relationship, when comparing the factors of the PMHQ construct and F2 (Prosocial Attitude), F3 (Self-Control), F4 (Autonomy), and F5 (Problem-Solving and Self-Improvement), a positive correlation was observed with F2: Humor as a mechanism for controlling situations within the MSHQ construct. Other factors within the MSHQ showed no significant correlation with PMHQ scores (Table 6; Figure 1).

**Table 6:** Analysis of the correlation between MSHQ and PMHQ.

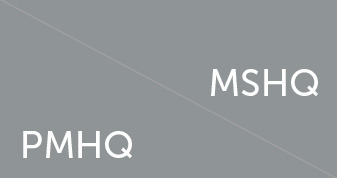	**F1: Personal competence or ability to use humor**		**F2: Humor as a mechanism for controlling the situation**		**F3: Social appraisal and attitudes toward humor**		**MSHS total**	
	***r***	***p***	***r***	***p***	***r***	***p***	***r***	***p***
F1: Personal satisfaction	−0.061	0.487	0.087	0.326	−0.091	0.301	−0.029	0.742
F2: Prosocial attitude	−0.093	0.290	0.264^*^	0.002	−0.099	0.265	−0.063	0.48
F3: Self–control	0.003	0.975	0.443^*^	0.000	−0.143	0.104	0.038	0.666
F4: Autonomy	−0.055	0.537	0.376^*^	0.000	−0.078	0.377	−0.038	0.664
F5: Problem solving and self-realization	0.029	0.746	0.343^*^	0.000	−0.159	0.070	0.062	0.487
F6: Interpersonal relationship skills	−0.098	0.267	0.084	0.345	−0.119	0.176	−0.050	0.576
PMHQ Total	−0.088	0.320	0.489^*^	0.000	−0.241	0.006	−0.017	0.844

MSHQ, Multidimensional Sense of Humor Questionnaire; PMHQ, Positive Mental Health Questionnaire; *r*, Pearson correlation coefficient; *p*, level of significance.

^*^Significant correlation.

## Discussion

The study found that mental health professionals generally had high levels of SH and PMH. While no overall positive correlation was seen between the two questionnaires, specific factors did show a positive relationship. Autonomy and humor appeared to be linked to certain sociodemographic and occupational traits, though these connections are complex and need more research.

Regarding the MSHQ, the average score for mental health care professionals in this study was generally 67.3 points. The global minimum score was 42 points and the maximum was 91. Although there are no standards with which to evaluate these results, considering that the global scores on the MSHQ ranged from a minimum of 0 to a maximum of 96 points, it can be safely stated that the professionals in the study exhibited moderately high levels of SH.

As for the PMHQ, the average score for mental health care professionals in this study is similar to that obtained for the same questionnaire in a study with mental health care professionals at Parc Hospitalari Martí i Julià in Salt, Girona (27), where the level of PMH received an average global score of PT: 97.7 points in 2015. The global minimum score was 71 points with a maximum of 112 according to reports on the study population. In this study, health care professionals achieved an average global score of PT: 102.6 points, with the minimum global score being 89 and the maximum 135, showing that health care professionals scored high in PMH.

These results indicate that health care professionals are more capable of feeling positive emotions than negative ones. Regarding the relationship between the PMH and SH variables for the professionals in the study, age turned out to be a variable not related to the sense of humor and was only significant in the F4: PMH Autonomy factor. This coincides with the data obtained by Mantas Jiménez et al. (27), where an increase in age showed higher levels of PMH and a greater ability of health care professionals to maintain their own criteria, be independent, have self-control over their own behavior, and have confidence in themselves. As for the professional category, both for the MSHQ and PMHQ, psychiatrists tend to have high scores in the F1 factor (Ability to use humor) and the F4 factor (Autonomy) compared to all other professional categories. It is worth noting that the TCAI speciality also presents equally high scores as psychiatrists in PMHQ, thus demonstrating their ability to maintain relative serenity and independence and, consequently, facilitate the self-regulation of behavior and confidence. In this study, the greater the experience in years in the field of mental health care, the more positive is the trend on the MSHQ regarding the F2 factor: Humor as a mechanism to control.

Humor offers some control within the patient-health professional relationship, with a greater predisposition to identify humor and appreciate it as useful. In PMHQ factors F3: Self-Control and F5: Problem-Solving and Self-Improvement, concerning years of experience in mental health care, professionals scored higher. There is a more positive trend for professionals with more years of experience who show a greater ability to face conflict and stress situations and possess better analytical and decision-making skills. Similarly, Fischer et al. (28) assert that possessing SH should be considered a healthy and desirable personality trait. This attribute correlates with the ability to respond positively in serious, uncomfortable, or stressful situations. Humor is seen as a personal quality that promotes resilience and emotional wellbeing. In summary, this allows professionals to be more flexible and have a greater capacity to adapt to changes occurring within the therapeutic relationship. Meanwhile, in the MSHQ, higher scores are obtained in contract variability for those with more stable contracts compared to those with substitute contracts, highlighting a higher score in factor F1: Personal competence or ability to use humor.

This suggests that professionals with greater job stability use and generate humor in their social lives and possibly in their relationships with patients and families. Another study (29) concludes that the workplace is a key environment that affects the mental health and wellbeing of working adults. To promote and foster mental health, workplaces should consider the importance of psychosocial wellbeing and the general health of staff, while providing an environment that supports and maintains both health and work efficiency. More detailed studies are now required to explore these findings more deeply.

### Limitations and practical application

This study has several limitations, including the composition of participants, which is not representative of the population of Catalonia. It is essential to replicate this study in other populations that reflect more diverse professional contexts to confirm the relationship between sense of humor (SH) and positive mental health (PMH) in mental health professionals. Moreover, to evaluate the consistency of results over time and understand how SH may influence PMH, rigorous longitudinal study designs are recommended. Another significant limitation is the lack of a gender perspective in the analysis of the studied variables.

SH and PMH are fundamental components within the field of mental health care, as they play a crucial role in professionals' ability to provide more competent and humane care. The methodological tools applied in this study enable mental health professionals to deepen their understanding, aiming to develop strategies that improve communication with patients and their families, offering closer and more personalized care, while also helping to reduce the stigma associated with mental health diagnoses.

The relevance of these findings to clinical practice lies in the potential to implement interventions that promote a healthier work environment for mental health professionals, which could positively impact their wellbeing and, consequently, the quality of care they provide. The fact that SH and PMH levels may be linked to sociodemographic and occupational characteristics suggests that implementing targeted programmes to enhance these aspects could have a significant impact on clinical practice.

Finally, this study lays the groundwork for future research, including multicentre studies that verify SH and PMH levels and explore the potential existence of a positive relationship between both scales, using a larger and more representative sample. The need for further research in this area is evident, given the potential impact on the mental health of healthcare workers.

## Conclusions

In general, the sample of mental health professionals in this study exhibited high levels of SH and PMH. Although no general positive correlation was observed between the two questionnaires used, a positive relationship was identified in certain specific factors. The results indicate that both the level of autonomy and the use of humor may be related to certain sociodemographic and occupational characteristics; however, the nature of these relationships remains complex and requires further investigation.

The study's findings suggest that mental health service administrators play a key role in improving the work environments of professionals, which may contribute to reducing stress and increasing self-esteem. Additionally, actively promoting workers' health not only enhances interactions with patients and their families but also strengthens the therapeutic relationship by facilitating more empathetic, personal, and genuine interventions. These findings highlight the importance of implementing strategies aimed at improving the mental health of mental health professionals within institutions, in order to ensure a healthy and sustainable work environment in the long term.

## Data Availability

The raw data supporting the conclusions of this article will be made available by the authors, without undue reservation.

## References

[1] Tolosa-Merlos D Moreno-Poyato AR Delgado-Hito P. The therapeutic relationship as the core of nursing care in acute mental health units: an analysis of the context in Catalonia. Cult Care. (2021) 25. 10.14198/cuid.2021.59.14

[2] Moreno-Poyato AR Montesó-Curto P Delgado-Hito P Suárez-Pérez R Aceña-Domínguez R Carreras-Salvador R. The therapeutic relationship in hospital psychiatric care: a narrative review from the perspective of nurses and patients. Arch Psychiatr Nurs. (2016) 30:782–87. 10.1016/j.apnu.2016.03.001 27888975

[3] Tizón García J. Humour in Interpersonal Relationships. Barcelona: Herder Editorial (2005).

[4] Carvalho JC Cordeiro R Martinho J Melo Tavares CM Postigo Mota S. Humour as a resource against stigma in mental health. Nurs ROL J. (2019) 42:286–92.

[5] Chelly F Kacem I Moussa A Ghenim A Krifa I Methamem F. Healing humour: the use of humour in the nurse-patient relationship. Occup Environ Med. (2022) 10:217–31. 10.4236/odem.2022.103017

[6] Pazargadi M Fereidooni Moghadam M Fallahi Khoshknab M Alijani Renani H Molazem Z. The therapeutic relationship in the shadow: nurses' experiences of barriers to the nurse-patient relationship in the psychiatric ward. Issues Ment Health Nurs. (2015) 36:551–57. 10.3109/01612840.2015.1014585 26309175

[7] Lluch-Canut MT. Promoting mental health in nursing care. Presence. (2005) 1.

[8] Sequeira C Carvalho JC Sampaio F Sá L Lluch-Canut T Roldán-Merino J. Evaluation of the psychometric properties of the positive mental health questionnaire in Portuguese higher education students. Rev Port Enferm Saúde Ment. (2014) 45–53. Available at: http://hdl.handle.net/10400.14/19775

[9] Teixeira S Sequeira C Lluch T. Positive Mental Health Promotion Program for Adults (Mentis Plus+): Support Manual. Barcelona: University of Barcelona Digital Repository, OMADO Collection (2021).

[10] Carbelo B. Humour in the Patient Relationship: A Guide for Health Professionals. Barcelona: Elsevier Masson (2005).

[11] García-Moran MC Gil-Lacruz M. Stress in the field of health professionals. Persona. (2016) 19:11–30. 10.26439/persona2016.n019.968

[12] Godoy D Eberhard A Abarca F Acuña B Muñoz R. Psychoeducation in mental health: a tool for patients and families. Clin J Las Condes. (2020) 31:169–73. 10.1016/j.rmclc.2020.01.005

[13] Cuevas-Cancino JJ Moreno-Pérez NE. Psychoeducation: nursing intervention for family care in the caregiver role. Univ Nurs. (2017) 14:207–18. 10.1016/j.reu.2017.06.003

[14] Lluch MT. Empirical evaluation of a conceptual model of positive mental health. Salud Ment. (2002) 25:42–55. Available at: https://www.redalyc.org/articulo.oa?id=58242505

[15] Peterson C Seligman ME. Character Strengths and Virtues: A Handbook and Classification. Vol. 1. Oxford: Oxford University Press (2004).

[16] Jáuregui E. The Sense of Humour: Instruction Manual. Barcelona: RBA Books (2018).

[17] Liptak A Tate J Flatt J Oakley MA Lingler J. Humour and laughter in persons with cognitive impairment and their caregivers. J Holist Nurs. (2014) 32:25–34. 10.1177/0898010113500075 23926217 PMC4006667

[18] Hirsch RD Junglas K Konradt B Jonitz MF. Humour therapy in depressed elderly: results of an empirical study. J Gerontol Geriatr. (2010) 43:42–52. 10.1007/s00391-009-0086-9 20143202

[19] Cai C Yu L Rong L Zhong H. Efficacy of humour intervention for patients with schizophrenia: a randomised controlled trial. Psychiatr Res. (2014) 59:174–8. 10.1016/j.jpsychires.2014.09.010 25266473

[20] Christie W Moore C. The impact of humour on cancer patients. Clin J Oncol Nurs. (2005) 9:211–8. 10.1188/05.CJON.211-218 15853164

[21] Parse RR. The experience of laughter: a phenomenological study. Nurs Sci Q. (1993) 6:39–43. 10.1177/089431849300600110 8455872

[22] Parse RR. Laughing and health: a study using Parse's research method. Nurs Sci Q. (1994) 7:55–64. 10.1177/089431849400700205 7808706

[23] Lluch MT. Construction of a Scale to Assess Positive Mental Health (Doctoral thesis). Faculty of Psychology, University of Barcelona, Barcelona, Spain (1999).

[24] Carbelo B. Validation of an Instrument to Measure Sense of Humour, Analysis of the Questionnaire and Its Relationship With Stress (Doctoral thesis). University of Alcalá, Madrid, Spain (2006).

[25] IBM Corp. IBM SPSS. Statistics for Windows. Version 24.0 [Computer Software]. Armonk, NY: IBM Corp (2016).

[26] Cuschieri S. The STROBE guidelines. Saudi J Anaesth. (2019) 13:S31–4. 10.4103/sja.SJA_543_18 30930717 PMC6398292

[27] Mantas Jiménez S Juvinyà i Canal D Bertran i Noguer C Roldán Merino J Sequeira C Lluch Canut M. Evaluation of positive mental health and sense of coherence in mental health professionals. Port J Mental Health Nurs. (2015) 34–42. Available at: http://hdl.handle.net/2445/144634

[28] Fischer F Peifer C Scheel T. Interdisciplinary perspectives on the relationship between humour and health: theoretical foundations, empirical evidence and implications. Front Public Health. (2021) 9:774353. 10.3389/fpubh.2021.774353 34760867 PMC8572968

[29] Picco L Yuan Q Vaingankar JA Chang S Abdin E Chua HC. Positive mental health among health professionals working at a psychiatric hospital. PLoS ONE. (2017) 12:e0178359. 10.1371/journal.pone.0178359 28591203 PMC5462373

